# Targeting oral cancer epigenome via LSD1

**DOI:** 10.18632/aging.101343

**Published:** 2017-12-11

**Authors:** Manish V. Bais

**Affiliations:** Department of Molecular and Cell Biology, Boston University Henry M. Goldman School of Dental Medicine, Boston, MA 02118, USA

**Keywords:** LSD1, epigenetics, oral cancer, OSCC mechanism

Oral squamous cell carcinoma (OSCC) accounts for the majority of head and neck cancers. Treatment for OSCC frequently comprises a combination of surgery, radiotherapy, and chemotherapy. However, resistance to therapy complicates treatment, and the 5-year survival rate remains at ~65 percent. Understanding contributors to disease progression and treatment resistance is needed to promote patient outcomes. Lysine-specific demethylase 1 (LSD1) is an amine oxidase with demethylase activity and has been implicated in maintaining the undifferentiated state of cancer-initiating cells. We have determined that lysine-specific demethylase 1 (LSD1) promote growth and metastasis of human and mouse OSCC [1, 2].

LSD1 expression is elevated in clinical OSCC compared to dysplastic and hyperplastic tissue specimens, and is absent in adjacent normal tissues. In a tissue microarray containing a diverse population of 80 OSCC (different grade/stage) of the larynx, tongue, and submandibular gland, LSD1 staining positively correlated with disease grade. Indeed, bioinformatics analysis of mRNA expression data from The Cancer Genome Atlas (TCGA) (from more than 300 OSCC) confirmed that LSD1 expression increases with tumor stage and grade.

We have established OSCC mouse models for understanding the basic mechanism and therapeutic preclinical applications [1-5]. We found that LSD1 knockdown in implanted HSC-3 orthotopic tumors attenuates tumor growth and metastasis, whereas over-expression of LSD1 promotes disease progression. Further, small molecule inhibitors (e.g., GSK-LSD1) of LSD1 attenuate disease progression, EGFR-induced signaling, and tumor-promoting gene expression (MMP13, LOXL4, and CTGF) in patient-derived xenografts. Microarrays followed by gene set enrichment analysis also showed that GSK-LSD1 inhibits key mediators of OSCC [1].

LSD1 has a dual and context-dependent role in transcriptional regulation Notch signaling [6] and regulation of androgen receptor in prostate cancer [7]. LSD1 can demethylate H3K4 during gene repression and H3K9 during gene activation. LSD1 demethylates histone and non-histone genes by removing mono- and dimethyl groups from histone H3 at lysine 4 (H3K4me1/2) without affecting trimethylation. Inactivation of LSD1 promotes G1 arrest and induces differentiation-specific genes by selectively modulating the methylation states of H3K4 and H3K9. Thus, expression of LSD1 may enable epigenetic regulation of its targets, and identifying LSD1-regulated molecular signaling mechanisms could reveal new targets for OSCC therapy. We recently implicated LSD1 in a novel action with the Hippo signaling effector Yes-Associated Protein (YAP), EGFR, NF-kB, and epithelial-mesenchymal transition in OSCC. GSK-LSD1 blocks YAP-induced oncogenic signaling pathways in patient-derived tonsillar epithelial, myoepithelial, and osteosarcoma tumor cells. YAP and TAZ, key effectors of the Hippo pathway, drive pro-tumorigenic signals in OSCC [5]. GSK-LSD1 inhibits YAP-activated down-stream targets such as SERPINE1 and CTGF in microarray analysis. However, which genes are functional targets of LSD1 and exert the effects on these signaling pathways—ultimately promoting OSCC progression and metastasis—is not known.

EGFR signaling is implicated in OSCC progression metastasis and therapeutic resistance. Inhibition of LSD1 attenuates proliferation and EGF-induced signaling, phospho-AKT, and ERK1/2 as well as NF-kB and its associated transcriptional networks. Further, GSK-LSD1 attenuates NF-kB signaling, which is implicated in inflammatory signaling pathways and checkpoint regulation. Although GSK-LSD1 attenuates NF-KB signaling, whether it attenuates downstream inflammatory effectors is not known.

**Figure 1:**
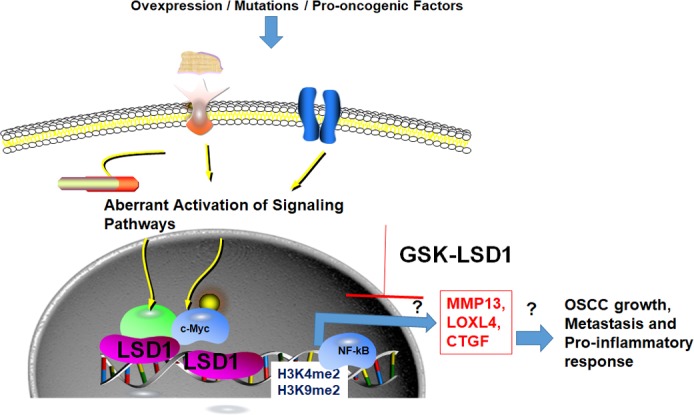
The potential role of LSD1 growth, metastasis, and pro-inflammatory response to OSCC Our studies showed that LSD1 expression or recruitment to chromatin is induced by EGFR- or YAP-induced signaling pathways. LSD1 may induce demethylation of dimethylated H3K4/H3K9 or alter NF-kB signaling, resulting in expression of MMP13, LOXL4, and CTGF. The coordination of different signaling pathways regulated by LSD1 could lead to OSCC growth, metastasis, and a pro-inflammatory response. An LSD1 inhibitor (GSK-LSD1) that attenuates LSD1 function could thereby inhibit OSCC. Unknown roles are indicated by (?).

Taken together, these results led us to ask the following questions (Figure 1): Is LSD1 a key mediator of the pro-inflammatory response in OSCC? Does LSD1 regulate OSCC-specific mechanisms, and if so, can it be exploited for monotherapy or combination therapies? Our long-term objectives are to understand the LSD1-induced molecular mechanism(s) underlying OSCC growth and metastasis and to identify its therapeutic potential for this disease. Epigenetic inhibitors that could reset the OSCC epigenome may synergistically correct deleterious effects induced by somatic mutations or oncogenic pathways. Currently, various LSD1 inhibitors, including GSK-LSD1 (GlaxoSmithKline) [8], are in phase 1 clinical trials for other cancer. LSD1 induced novel OSCC specific mechanisms, chemical and biologic inhibitors under development to interfere aberrant biological function of LSD1 could be applied to OSCC therapy. Epigenetic targetting of OSCC via LSD1 may have broader implications for other tumor types and translational studies.
